# Automated facial feature evaluation system to prevent stress of head fixed mice

**DOI:** 10.1371/journal.pone.0322530

**Published:** 2025-06-23

**Authors:** Anna Nasr, Gianna Rettinger, Huibert D. Mansvelder, Robert N.S. Sachdev, Matthew E. Larkum

**Affiliations:** 1 Institut für Biologie, Neurocure Center for Excellence, Charité Universitätsmedizin Berlin and Humboldt Universität, Berlin, Germany; 2 Vrije Universiteit Amsterdam, Amsterdam, Netherlands; 3 Technische Universität Berlin Institut für Biotechnologie, Berlin, Germany; Kyoto University Graduate School of Informatics: Kyoto Daigaku Daigakuin Johogaku Kenkyuka, JAPAN

## Abstract

Head fixation of rodents is a widely utilized and important technique that enables laboratories to measure brain activity during behavior, but head fixation can increase stress which affects both behavior and underlying brain activity, as well as animal welfare. It is therefore critical to keep stress levels low, yet it is particularly challenging to assess stress in immobilized, head-fixed rodents. Conventional stress evaluation methods, such as blood corticosterone analysis, are labor-intensive and conducted post hoc, and In situ approaches require experienced personnel and constant vigilance during experiments. In this study, we present MouseCare, an automated software solution for immediate stress detection by real-time facial feature video analysis in head-fixed mice. MouseCare performs on par with or better than conventional stress measures, and is significantly less labor-intensive. This approach enables objective measures of stress that are needed to determine when an experiment can commence, but also when it should be stopped. We conclude that MouseCare offers a cost-effective and easily implementable strategy to manage stress levels in rodents that can increase data quality and improve animal welfare.

## Introduction

Head fixation is a technique widely used in neuroscience laboratories to immobilize the head of an experimental animal [1–8], facilitating various essential experimental approaches [9–16], including 2-photon imaging [17] and patch-clamp recordings in vivo [18]. To immobilize the head, a fixed head-post is secured to the animal’s skull to restrict head movement. While head fixation is invaluable for scientific research, prolonged immobilization can be stressful, impacting their welfare and experimental outcomes [19–22]. Additionally, it can be expensive and time-consuming, requiring specialized equipment and skilled personnel. Therefore, it is essential to constantly monitor stress levels in mice to ensure their well-being and the reliability of experimental results. Balancing scientific needs and animal welfare is crucial [23].

The first step to mitigating stress is early detection. Habituation and training are necessary for animals to become stress-free under head fixation. Familiarizing the animal with the setup and tasks before recording can help reduce stress [24], but objective assessment of when a mouse is habituated is challenging. A method to systematically and objectively track stress during habituation is important for optimizing the well-being of animals undergoing this procedure.

Detecting stress in head fixed mice is critical for accurate scientific research. Among various methods, measuring stress hormone levels in blood via tail vein is the gold standard due to its precision. However, this method presents a paradox. The procedure of blood sampling induces mild to moderate stress in animals [25–27]. Thus, the most accurate method to detect stress—blood sampling—inevitably is invasive and causes stress. This contradiction poses a significant challenge, as the act of measuring stress can alter the very parameter being measured. Therefore, there is a pressing need to develop an accurate, real-time, non-invasive stress detection method to reduce experimental stress.

One such alternative is the analysis of corticosterone metabolites in feces, which offers a non-invasive approach to stress detection. While this method significantly reduces the stress associated with sample collection, it comes with its own set of challenges, particularly in terms of accuracy. The levels of corticosterone metabolites can be influenced by various factors, including the daily rhythm (circadian cycle), sex, age, and individual metabolic differences. These influences can introduce variability and complicate the interpretation of results. Additionally, corticosterone metabolite analysis requires collecting and processing stool samples, which introduces a significant time lag between the occurrence of stress and the detection of stress [28–31]. This delay is acceptable when assessing overall stress levels in an environment over time, but incompatible with the need for real-time stress monitoring.

Analyzing stress indicators in urine [32], saliva [33], or milk [34], have also been explored. However, these methods often require extra equipment to extract the samples, adding complexity and potential stress to the process. Despite these limitations, corticosterone metabolite analysis represents a promising step towards balancing the need for reliable stress measurement with the imperative to minimize animal distress.

In addition to biochemical methods, observational techniques offer a valuable means of assessing stress in real-time. Researchers can utilize the Mouse Grimace Scale, a validated tool for identifying pain and distress through facial expressions, to evaluate stress on the fly [35–37]. This method allows for immediate assessment and intervention, enhancing the welfare of laboratory animals, without the need for invasive procedures or complex sample analysis. However, the accuracy of this observational approach heavily relies on the skill and experience of the researcher, which can introduce variability and potential bias into the stress assessment [37,38]. Moreover, researchers are typically unable to simultaneously attend to ongoing experimental needs and assess stress.

Recent advances in artificial intelligence, such as deep neural networks, have provided new opportunities for detecting stress in head fixed mice. A deep neural network is a type of machine learning algorithm that can analyze large amounts of data and identify patterns and relationships between variables.

These networks can be trained to track individual pre-defined points in a video, an example of which is DeepLabCut [39]. Using this as a basis, it is possible to track and evaluate changes in different facial features (similar to how the grimace scale works). This information can then be used to update the researcher during the experiment on the well-being of the head fixed mice, independent of the researcher’s experience.

Overall, detecting stress in head fixed mice is crucial for ensuring their welfare and the validity of scientific experiments. Therefore, we have developed MouseCare, a cost-effective and easily implementable strategy to evaluate stress levels in head fixed mice.

## Materials and methods

All experiments were conducted in accordance with the guidelines of animal welfare of the Charité Universitätsmedizin Berlin and the local authorities (Landesamt für Gesundheit und Soziales, LAGeSo). All mice underwent surgery at the beginning of the experiment.

### Surgery

Adult C57BL/ 6 mice (n = 24) were anesthetized with Ketamine/Xylazine (95 mg/ kg and 5 mg/ kg), positioned in a stereotactic apparatus (David Kopf Instruments, California, US) and placed on a heating pad (FHC Inc. Maine US). During surgery, the eyes were covered with ointment (Bepanthen, Bayer, Leverkusen, Germany) to prevent them from drying out. Before making an incision, Lidocaine was injected under the skin and the scalp was disinfected with ethanol. The skull was exposed, the fascia on the bone scraped off with a dental scraper and the skull was air dried. A light-weight aluminum head post was laid on the skull and RelyX (3M, Minnesota, US) cement was used to affix the head post to the skull [40–42]. Black jet acrylic (Ortho Jet, Lang Dental) was used as a second layer to cover the exposed bone and to enhance the cementing of the head post. For analgesic purposes, Buprenorphin (0.1 mg/kg) was injected IP on the day of surgery and Carprofen (5 mg/kg) was injected IP on the day of surgery and for two days post surgery.

In addition, we received video footage of head fixed mice from other projects. This allowed us to evaluate our system with different types of head fixed experiments, without requiring additional mice. The corticosterone metabolite analysis of our stool samples was performed by Prof. Dr. R. Palme from the Vetmeduni Vienna Unit of Physiology, Pathophysiology and Experimental Endocrinology.

### Tracking facial features with MouseCare

**Fig 1:**
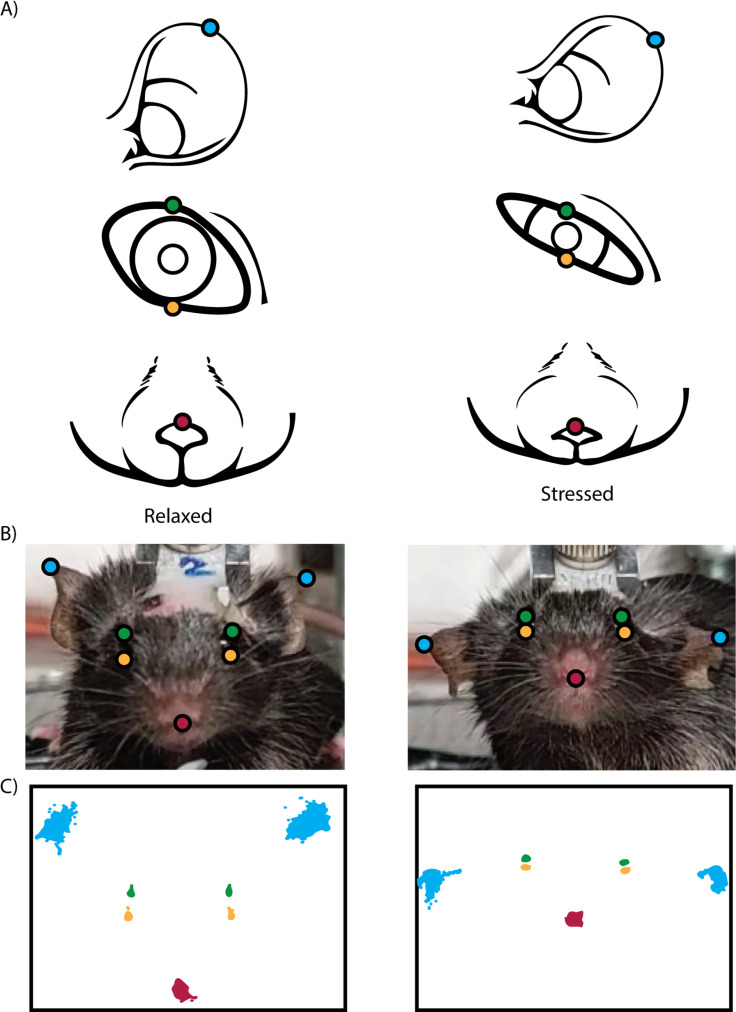
Tracking the facial features of a head fixed mouse. A) Illustration of the different facial features that are tracked and their change and positional change from relaxed to stressed. B) Screen capture of Video footage from a camera facing head fixed mouse with the tracking points overlaid. C) Positional frame summary of the movement range for each facial feature.

The automated stress detection, with MouseCare(https://github.com/Nasr-SFB1315/MouseCare), uses pre-processing by DeepLabCut (DLC) to track specific facial features of head fixed mice. DLC is an open-source toolbox for detecting features using deep learning that is particularly useful for tracking moving body parts in rodent experiments [39]. In this study, we created a neural network (using the DLC toolbox) and trained it on the facial features of head fixed mice. To do so, we used 24 different 5-minute Videos and extracted 12 frames per video. The training was done after 180000 iterations. Additional setups with different lighting required 2–3 extra videos to further train the network on the new environment. We labeled seven distinct facial features on a set of training images: each ear, each upper and lower eyelid, and the nose (Fig 1A). These features were chosen on the basis of their efficacy for detecting different stress levels and their distinctiveness due to movement, size, and position (versus other features such as cheeks, whiskers, etc. which we found were not as useful for stress detection). The network was trained to accurately identify these features in a variety of video frames, achieving an accuracy rate of 99% (Fig 1B and C). Further examples from different setups are provided in (S1 Fig). This high level of accuracy ensures reliable tracking of these features across different video sessions. Once the network training was completed, we applied it to pre-recorded videos to analyze the position of each labeled facial feature over time (Fig 1C). The coordinates of these features were extracted and stored in csv or h5 file formats for further analysis. The stability of MouseCare depends on the balance between camera hardware and computational capacity. While higher frame rates and resolutions can provide more detailed data, they also generate larger data loads that may overwhelm the processing capabilities of the system. We used a frame rate of 30–60 FPS. This was sufficient for tracking facial features accurately in our setups. While higher frame rates (e.g., 200 FPS) are technically feasible, they significantly increase the data load and may cause performance issues depending on the computer’s hardware. The resolution of 1080p (1920x1080 pixels) was sufficient for accurate tracking using colored cameras. Similar to the frame rate, higher resolution were possible but not feasible. We tested different cameras and lighting in the setups. Screen captures of those recordings are shown in (S1 Fig).

### MouseCare system for stress detection

We developed a Python-based software, to perform the stress detection assessment using our facial feature tracking network (developed with the DLC toolbox) with heuristic based algorithms. This complete system we call MouseCare. The customized software component of MouseCare was designed to analyze the positional data of the tracked facial features and detect stress in the mice. To achieve this, we designed MouseCare to integrate the coordinate data and apply a series of heuristics to assess stress-related behaviors (see below). Here, using the processed DLC output, the rest of the system identifies patterns and indicators of stress, leveraging behavioral analysis techniques, and statistical analyses to provide robust and reliable stress detection. The results could then be compiled and visualized for interpretation.

The individual positions of each facial feature (Fig 1A) as well as the movement of these features were crucial for the analysis and were evaluated over specific periods (epochs) to exclude false positives. Squinting eyes are a known sign of stress and discomfort [43], but can also be triggered by other factors. For instance, in a previous experiment, we observed squinting and rapid blinking when mice received a liquid reward from a licking port [24]. We distinguished these types of squinting by their duration: reward-induced squinting lasted less than one second, while stress-induced squinting persisted for extended periods. This is achieved by calculating the Euclidean distance ($\begin{array}{c} d\;=\;\sqrt{}[{\left(x2\;–\;x1\right)}^{2}+\;{\left(y2\;–\;y1\right)}^{2}] \end{array}$) between the upper and lower eyelid while averaging the x and y over the fps. These values are stored in a dataframe. Subsequently, the dataframe is analyzed to count consecutive entries where the eyelids remain close together, excluding rapid blinking.

Freezing is another stress response. To measure it, we calculated the positional changes of different facial features (ears and nose) over a 2-second time span. Rodents are able to move their nose in 3 dimension while head-fixed [44]. Using a front-facing camera, we recorded nose movements along the left/right (X) and up/down (Y) axes, storing positional data for each frame over 2 seconds. From this data, we calculated the Maximum Range of Motion (The largest displacement of the nose during the 2-second window) and the total Nose Travel Distance (The cumulative Euclidean distance between consecutive nose positions) These metrics were combined to compute the Nose Activity Index, a quantitative measure of nose movement. The same methodology was applied to the ears. In addition to tracking ear movement, we compared positional data between the two ears to detect asymmetry, as unilateral ear movement can indicate discomfort. Finally, we used the maximum range of motion to determine if an ear was folded to one side, another potential indicator of stress.

### Live stress detection with MouseCare

**Fig 2:**
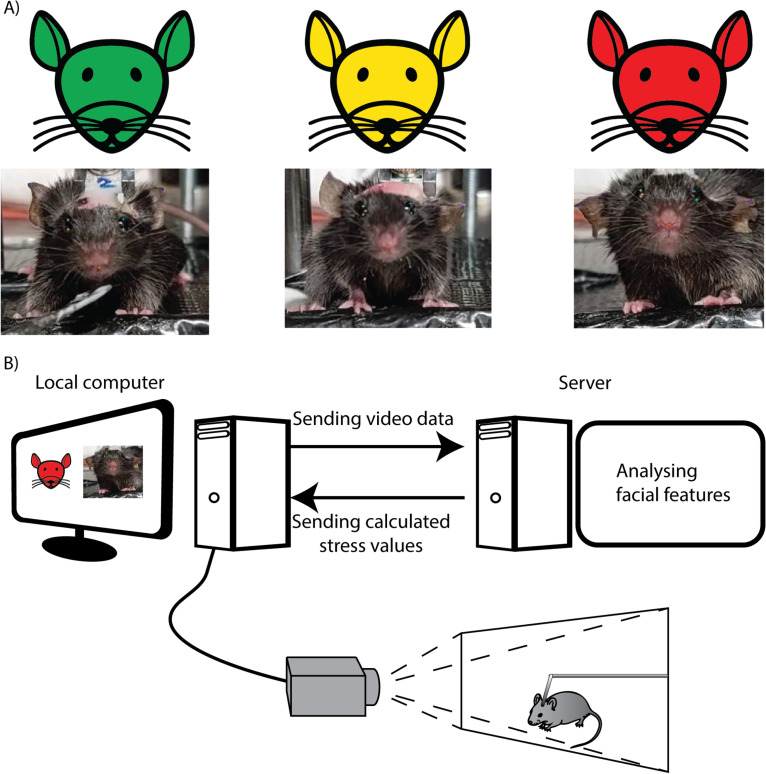
Schematic of the live video transfer from the experimental computer to the analysis computer and the return of the stress evaluation results. A) Depiction of the 3 different stages of stress shown via video capture and the corresponding icon. B) Schematic representation of the camera setup and the data transfer between the experimental computer and analysis computer.

Our goal was to generate a user-friendly system for live feedback on the stress state of the mouse, prioritizing the ability of the researcher to react immediately to ongoing changes in stress levels. To achieve this, we used the open source toolkit DeepLabCut Live (DLC Live) [45], which used our pre-trained DLC deep neural network, to pipeline the detected coordinates to MouseCare. The MouseCare software continuously evaluated the stress of the mouse according to the stress heuristics on a scale of 0–10. To facilitate easy interpretation of stress levels during experiments, MouseCare displays live stress levels with a colour-coded 3-value system (Fig 2A, okay/green, caution/yellow, stressed/red). Open Broadcaster Software (OBS) was used in conjunction with a local camera to stream video to an external computer (Fig 2B). This has the dual benefit of allowing for centralization of computer resources and also for remote monitoring. By sending only the current status back to the experimental computer, we drastically reduced the burden on local real-time computation.

### Importance of weight adjustment for facial feature analysis

**Fig 3:**
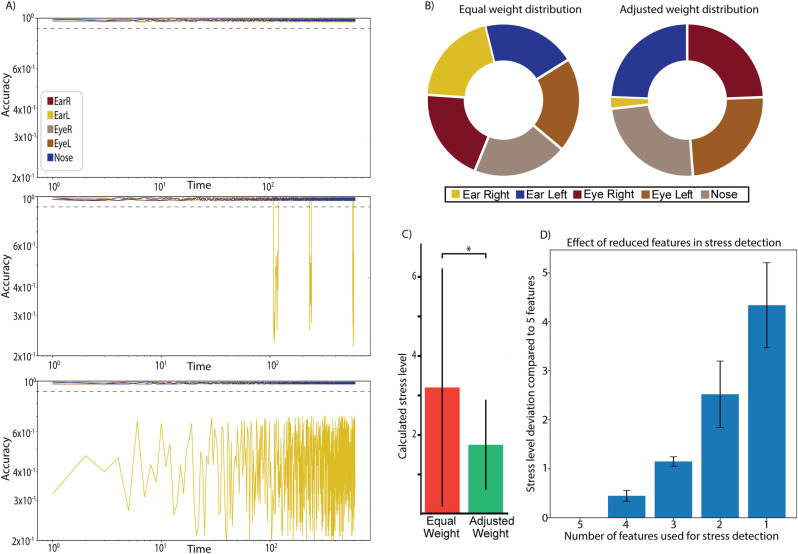
Evaluation of the effect of facial feature weight adjustment. A) Three Logarithmic graphs of the accuracy for each facial feature. Dotted line, accuracy threshold. The accuracy of the Ear Left, Eye Right, Eye Left and Nose are close to each other and overlap in the graph. The top graph represents a mouse with all facial features visible. The middle shows the effect of temporal obstruction, moving objects between the mouse and the camera. The bottom shows the effect of permanent obstruction of a facial feature. B) Comparing the standard weight distribution and the adjusted weight distribution. C) Calculated stress level of the same mouse using equal weight distribution and adjusted weight distribution. D) Effect of removing facial features on stress level evaluation. The Y-axis shows the deviation in stress level compared to the baseline using all five features.

The variability in head fixation setups, particularly head posts, can significantly impact the movement of facial features in mice, and may also obscure them to a camera. For that, it is important to determine the effect it can have on the stress evaluation. We have separated them into three different categories: All facial features are visible, temporarily obstructed facial feature and permanent obscured a facial feature (Fig 3A).

MouseCare was designed to be flexible across multiple systems, we implemented a dynamic weighting system, which allows the user to adjust the evaluation of facial features at the start of each session. This system ensures that re-training was not required for different head posts, which might obstruct facial features [1–8]. The visibility and tracking accuracy for each of our setup was tested and evaluated. This was tested in six different setups with different cranial attachments, four of which are shown in (S1 Fig). It is important to highlight that while in some setups, all facial features might be visible but unable to move naturally due to cranial implants. And therefore were treated the same way as obscured facial features. Initially, each facial feature was assigned a weight of 1. As an example, in one set up an ear was pushed down by part of the setup, reducing its weight (Fig 3B) leading to more stable evaluation with fewer fluctuations in the stress evaluation (Fig 3C). To further investigate the effect of obfuscation/ removal of facial features, we analyzed recordings from head-fixed mice (N = 3), setting the weight of individual facial features to 0 to simulate their removal from the evaluation. Using the stress evaluation with all five facial features as baseline, we determined how stress level estimations deviated with a decreasing number of remaining features (Fig 3D). The Y-axis in (Fig 3D) represents the absolute deviation from the baseline stress level (same units as in Fig 3C).

### Stool analysis for stress evaluation

To analyze the stool samples of mice during the experiment we kept the mice in single cages and on the day of measurement we replaced the bedding to remove any form of old stool in the cage. After the experiment, the mice were placed back in the cage and 8 hours later, the feces were collected and dried. The dried samples were sent to a laboratory for processing (Palme laboratory at the University of Veterinary Medicine, Vienna). The dried stool samples were diluted, and the corticosterone metabolites were extracted with 1 ml of 80% methanol for 50 mg of feces, or with 0.8 ml for smaller fecal amounts.

## Results

Our goal was to develop a software-based system (“MouseCare”) that could quickly, accurately and reliably assess stress in rodents under experimental conditions, providing a superior substitute for current methods. For brevity, we provide the detailed methods of the development and training of the MouseCare algorithm in Methods and Figs 1–3. This involved providing videos of head-fixed mice with a mixture of mild stress-inducing situations (loud noises, vibrations, etc.). Next, we assessed the performance of MouseCare by comparing the stress levels determined by MouseCare with both subjective and objective measures of stress.

### Comparison to subjective assessments

**Fig 4:**
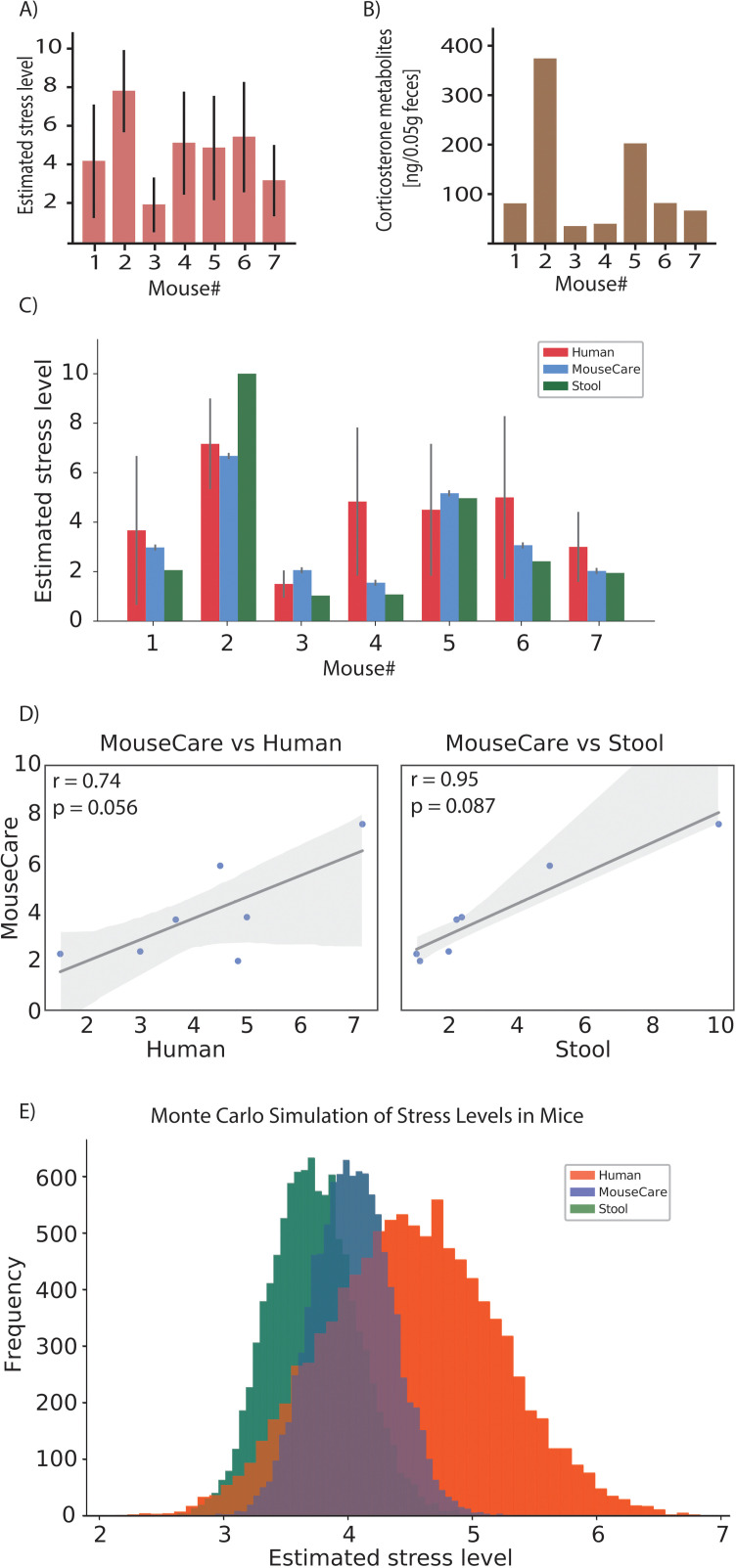
Comparing the stress evaluation results using different methods. A) Estimated stress level based on the facial feature evaluation of the participants. B) Measurement of the corticosterone metabolites for each of the mice shown in the 7 videos. C) Comparing the previous 2 methods with the results from MouseCare. Inter-rater reliability among the senior researchers was assessed using Cohen’s kappa coefficient to ensure consistency, and the Corticosterone metabolites were normalized. D)*Calculation of the Pearson correlation coefficients between the three methods for each mouse. The stool samples were normalized. The X and Y axis represent the stress level on a scale from 1-10. In gray is the confidence interval. The Pearson correlation coefficient is labeled “r” and the p value is labeled as “p”.* E) Using the Monte Carlo Simulation of the 7 mice and the three different methods of stress evaluation using 10000 simulations.

The vast majority of animal experiments use observer-assessed stress evaluation, in which facial features are the key indicators [43]. We therefore first tuned MouseCare based on stress-scores provided by trained scientists (n = 7). We first sought to evaluate how closely MouseCare tracked the assessment of typical stress behaviors compared to experimenters. To achieve this, we conducted a survey with two groups of participants: experienced researchers and inexperienced. The primary focus was on the experienced researchers’ assessments, while the results from the inexperienced group were included to provide a broader range of perspectives and are detailed in the supplementary materials (S1 Fig). Participants were presented with a series of 7-second video clips of head-fixed mice, without further information regarding the stage of habituation to head-fixation. Each participant independently assessed the stress levels of the mice based on observable facial features. Each participant rated the stress on a standardized scale from 1 to 10, with 1 indicating no stress and 10 indicating severe stress, in line with the scoring metric of MouseCare (Fig 4A).

### Comparison to objective assessments

To provide an observer-independent measure of stress, we analyzed corticosterone metabolites in stool samples (analyzed by Palme laboratory at the University of Veterinary Medicine, Vienna). The results were expressed as ng/50 mg of feces. The corticosterone metabolite levels varied, reflecting differences in habituation stages and experimental conditions (Fig 4B). It is important to mention that there are a lot of influences (daily rhythm, sex, age, diet, etc…) on fecal corticosterone metabolites (FCM) values [31]. MouseCare collected the positional data of each facial feature from the same videos as the participants, and then analyzed each facial feature individually and assigned a weighted stress score from 1 to 10 to each feature. Those individual stress scores were then averaged to generate a total stress level for each video. A final total stress average was calculated for the 7-second duration, this was done for each video individually. By comparing the three methods—manual observation by experienced researchers, corticosterone metabolite levels, and MouseCare—we found that all approaches yielded comparable stress evaluations (Fig 4C). Notably, the experienced researchers’ estimates tended to indicate the highest stress levels, while corticosterone metabolites suggested the lowest, with MouseCare in between. We quantified the consistency between the different approaches by measuring the Pearson coefficient (comparing MouseCare to human reports and MouseCare to FCM) (Fig 4D **additional comparisons are shown in** S3 Fig). Here, we found that MouseCare aligned closer to the stool sample with 0.95 compared to the survey 0.74.

We performed an additional analysis, we performed a Monte Carlo simulation (Fig 4E**, the full list of the parameters used for this simulation is in** S4 Fig) by randomizing the mice IDs for the MouseCare values (see Methods) to estimate the probability of obtaining profiles with this similarity. In general, the MouseCare results more closely followed the FCM estimates, suggesting that MouseCare and FCM are more consistent approaches than subjective human reports. A significant discrepancy was observed only in mouse 4, where the experienced researchers’ stress estimates were substantially higher than those indicated by corticosterone metabolites and MouseCare. The results from the inexperienced group were less self-consistent (S2 Fig), underscoring the need for efficient methods of stress evaluation.

### Effect of habituation and training on stress handling

**Fig 5:**
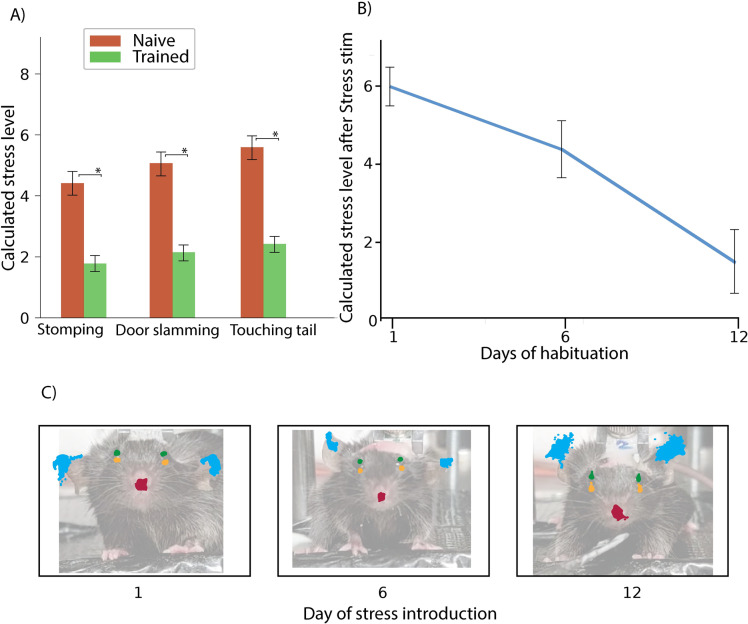
Impact of habituation on the response to external stress stimuli. A) Evaluation of the facial features between naive and trained mice in response to external stress stimuli using MouseCare. B) One time external stress stimulus after different days of habituation. C) Bar chart of the facial area of single mice at the day of stress introduction.

Our final objective was to evaluate the stress levels of head-fixed mice during different stages of their habituation, along with their responses to various external stimuli commonly encountered in laboratory settings, including loud noise, physical contact (e.g., touching the tail of the head fixed animal). By exposing the mice to these three distinct stimuli, we were able to discern their stress effects relative to habituation using MouseCare (Fig 5A).

The reactions of the mice to external stress events were assessed over time (Fig 5B) showing that the effect of the stress stimulus had decreased relatively to the amount of habituation the mice had. This provides valuable insights into the effect of habituation. The visual representation of the network tracking with the original mouse images underlaid displayed the changes in facial expression (Fig 5C) based on the amount of habituation.

## Discussion

In this paper, we presented a novel system to efficiently detect stress in head fixed mice. This approach prioritizes speed and accessibility over precision and includes easy-to-use custom-built software (MouseCare). Real-time facial analysis alerts researchers to signs of stress in mice with immediate feedback. Additionally, extraneous sources of stress (e.g., malfunctioning hardware causing irritating noises), can be identified, avoiding negative impact on behavior and training outcomes. We validated the performance of the software by comparing it with the experience of researchers as well as stool samples and showed that it performed equal-to-better compared to the other methods. The MouseCare software provides an overall stress score so that the researcher can easily react to, during the experiment. This approach simplifies stress detection in rodents, enhancing objectivity and reproducibility.

Stress detection in rodents has been relatively understudied. Automated systems using machine learning approaches that exist tend to focus on specific parameters relevant to particular behavioral paradigms [46–48]. MouseCare takes advantage of a preprocessing stage using a network made with DeepLabCut, that allows for body-part tracking for the algorithm to assess the stress. This makes the system more flexible and allows for relatively efficient adaptation to a given behavioral paradigm. For instance, this system could be easily adjusted to various setups and head fixation methods, utilizing its open-source nature to facilitate widespread adoption. The weight assigned to each facial feature allows for quick adjustments to accommodate different head posts or other types of obstructions. While this flexibility facilitates rapid adaptation, the removal of facial features impacts the accuracy of stress evaluation. Using five facial features as a baseline shown to provide the best stress evaluation results (Fig 4) we examined the effect of removing individual features to simulate obstructions (Fig 3D). Our findings indicate that the system remains reliable when a single facial feature is removed but becomes progressively less reliable as additional features are excluded. Therefore, it is recommended to test and optimize the system for each experimental setup to ensure consistent performance.

MouseCare was compared to both objective stress measures, e.g., corticosterone metabolites, and on subjective measures, i.e., human evaluation (Fig 4). To mitigate subjective bias, the human subjects used for the subjective assessment were experienced scientists (i.e., post-docs). As can be observed in Fig 4, the subjective and objective assessments were relatively close, and the training achieved AI-based assessments between these values. Note, subjective assessments could vary considerably based on the experience level of the researcher (S2 Fig), however we found that the experienced group was surprisingly consistent (with one outlying data point in Fig 4). The close correspondence of the assessments made by an objective measure (corticosterone metabolites) to the experienced researchers providesconfidence that MouseCare achieves the best possible AI-based score. Nevertheless, it should be noted that the most accurate objective measurement of stress is currently an analysis of levels of cortisol in the blood [25,26]. Corticosterone metabolites are used widely because of their convenience and the fact that they are non-invasive, however they have also been shown to have limitations, particularly regarding gender differences in mice [49]. In general, these objective approaches to stress detection can be difficult and time-consuming, and an automated approach will save researchers a lot of time and/or encourage more laboratories to use continual stress assessment for rodent-based studies. Even more importantly, the live automated approach with MouseCare, is essential for alleviating or mitigating the effects of stress during the experiment itself. An automated system such as MouseCare is also ideal for training new researchers. Here, we suggest that new researchers be trained on pre-evaluated video footage that can be evaluated readily with MouseCare. The use of video footage reduces the number of animals needed for training, which is beneficial from an ethical perspective. In many cases, it will be possible for trainees to use video of previous experiments on the same or similar systems, making the video assessment more relevant. After becoming proficient on videos, the new researcher can transition to live experiments under the continual assistance of MouseCare. Overall, we anticipate that an AI-onboarding process will significantly reduce the total stress for mice.

Our system’s ability to continuously monitor mouse facial features over extended periods also offers valuable insights into habituation on an individual basis. Environmental factors, such as laboratory noises, can vary between mice, setups, and experiments. This variability presents a significant hurdle in estimating the ideal duration for habituation in behavioral paradigms involving head fixation. MouseCare can be introduced at different points in the experimental timeline, depending on the researcher’s objectives. For individual animals, we recommend starting MouseCare during the first head fixation sessions, as this is when stress levels are typically highest, making early detection crucial for effective habituation. For researchers integrating MouseCare into their experimental protocols, however, we suggest implementing MouseCare after the initial head fixation stages, once mice are acclimated and can tolerate longer periods of fixation. This timeline allows researchers adequate time to calibrate and optimize the system without disrupting studies involving naive mice, which are limited to shorter fixation durations. Introducing MouseCare at this stage ensures the system is fully operational and provides accurate, continuous stress monitoring during critical phases of habituation and training. An AI-driven approach like MouseCare enables a more precise evaluation of habituation, maximizing benefits for the animal (in terms of stress reduction) and for the researcher (by minimizing habituation time). Furthermore, its open-source code allows for modifications and potential expansion to other species, such as rats, or even the detection of emotional states beyond stress, such as joy.

Cost-effectiveness is another crucial consideration. Unlike currently-available systems relying on expensive cameras and computers, MouseCare operates efficiently with more affordable equipment. We recommend using a high-end computer for operating MouseCare, however this can be accessed over a local network minimizing the need for high-end computers in each experimental room, thereby reducing overall costs. In the current design of MouseCare, we used affordable camera technology, however, we would expect potential improvements to pupil tracking and evaluation could be achieved with better cameras which might provide even greater accuracy in stress detection. Machine learning has also opened other avenues for stress detection, since it can also be used to evaluate other bodily functions like breathing [50]. This can be used in experiments where it is not feasible to have a camera recording the face of the mice.

In conclusion, our study demonstrates that MouseCare, an automated system for real-time facial feature analysis, effectively detects stress in head fixed mice. MouseCare performs as well as, or better than, traditional methods such as corticosterone metabolite levels and human observation. MouseCare has the steepest implementation hurdle compared to the other methods, but once it is adjusted to the setup it has the least amount of variability compared to the other two methods, making it more reliable. This system enhances the objectivity, reproducibility, and ethical standards of stress assessment in neuroscience research. By providing immediate feedback, MouseCare enables timely interventions, optimizing both animal welfare and experimental outcomes. Additionally, its adaptability to various experimental setups and cost-effectiveness make it a valuable tool for research laboratories. MouseCare represents a significant advancement in the field, offering a practical and efficient solution for continuous stress monitoring in rodent studies.

## Supporting information

S1 Fig Showing the tracking result of different mice in different setup with different lighting conditions. To test the versatility of MouseCare, we used different mice in different setup to test if the system is capable to track the facial features successfully under different conditions.
(TIF)

S2 Fig Comparing the results of the stress evaluation between the experienced and inexperienced group. The participants received 7 videos of head fixed mice and were tasked to evaluate the stress of the mice on a scale of 1 (no stress at all) to 10 (very stressed).
(TIF)

S3 Fig Calculation of the Pearson correlation coefficients between the three methods for each mouse. The stool samples were normalized. The X and Y axis represent the stress level on a scale from 1–10. In gray is the confidence interval. The Pearson correlation coefficient is labeled “r” and the p value is labeled as “p”.
(TIF)

S4 Fig Performance comparison of Stool samples, humans and MouseCare using a Monte Carlo simulation with additional Numeric values. A) Numeric results of the Monte Carlo simulation shown in Fig 4D. B) Visual representation of the Performance comparison three groups.
(TIF)
