# Supplementary figures and images for: Automated facial feature evaluation system to prevent stress of head fixed mice
Source: PLoS One. 2025 Jun 23;20(6):e0322530. doi: 10.1371/journal.pone.0322530 (PMC12184996; doi:10.1371/journal.pone.0322530)

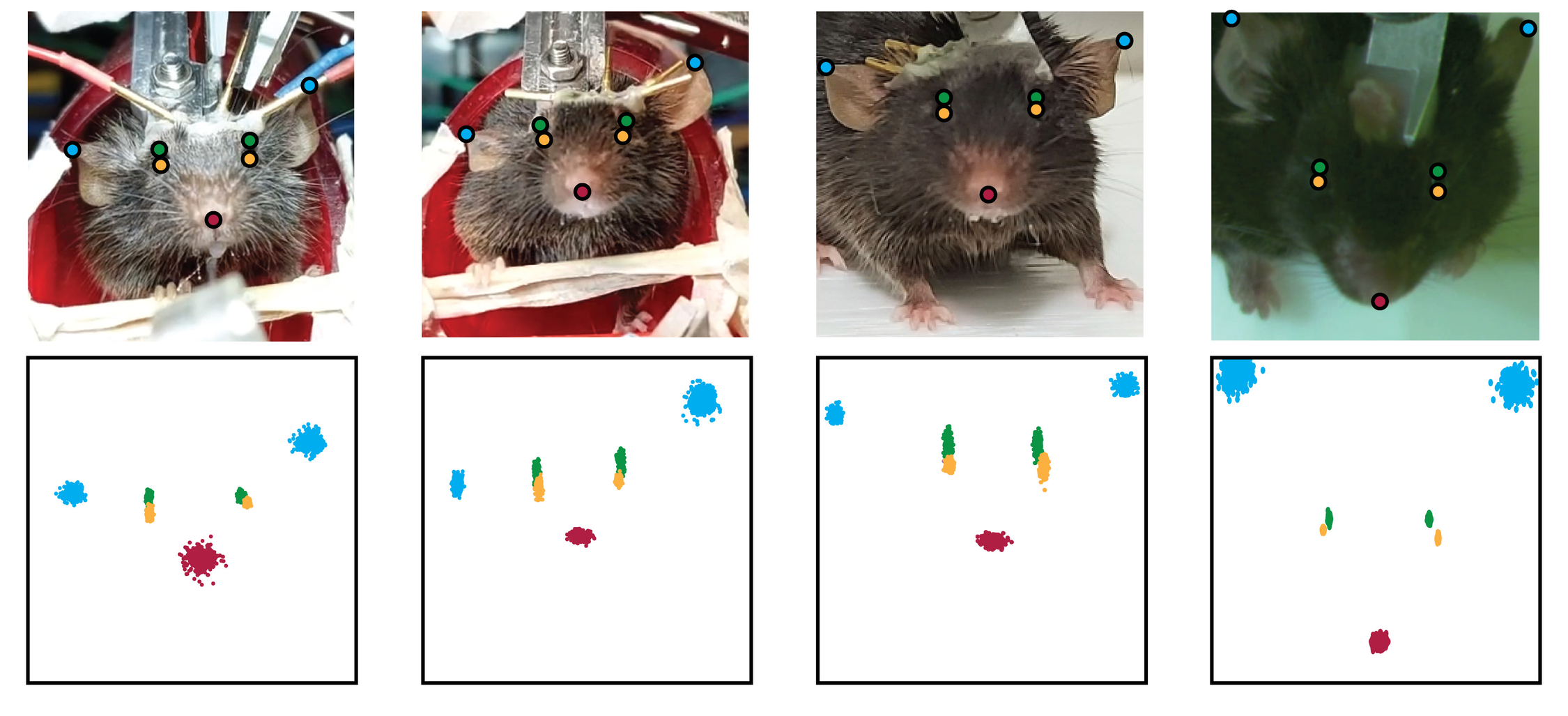

Supplement: S1 Fig — To test the versatility of MouseCare, we used different mice in different setup to test if the system is capable to track the facial features successfully under different conditions. (TIF) [file pone.0322530.s001.tif]

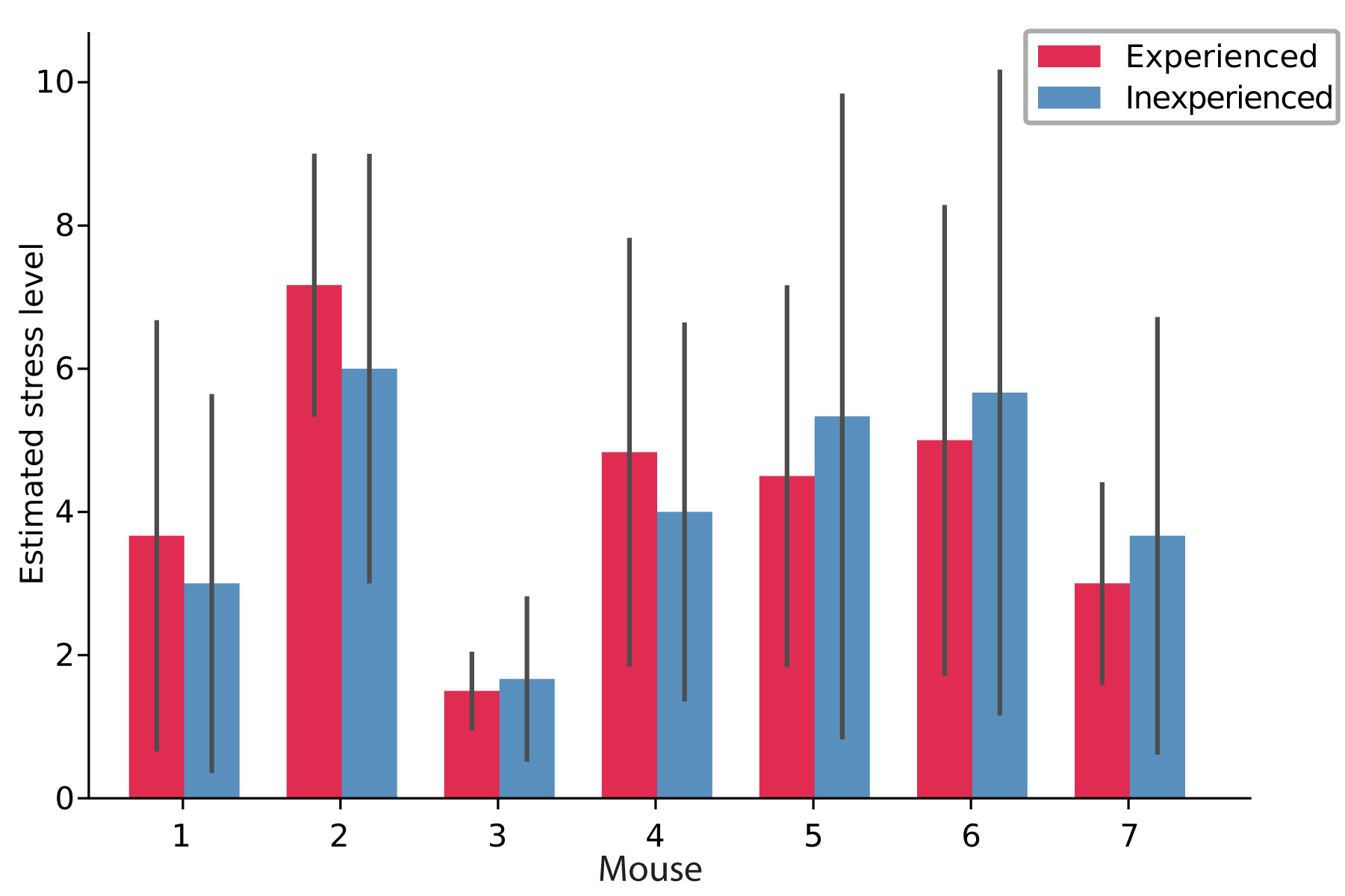

Supplement: S2 Fig — The participants received 7 videos of head fixed mice and were tasked to evaluate the stress of the mice on a scale of 1 (no stress at all) to 10 (very stressed). (TIF) [file pone.0322530.s002.tif]

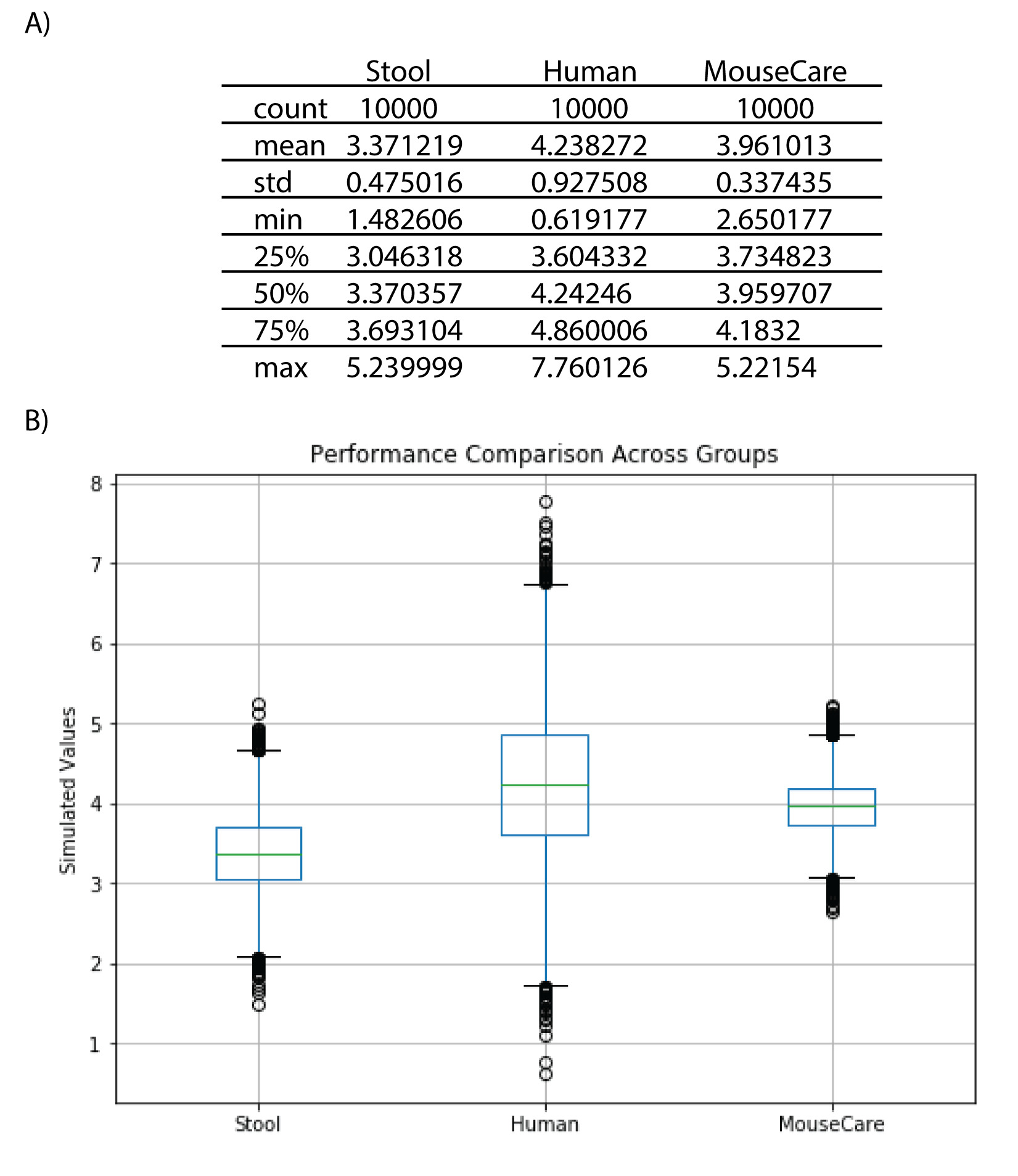

Supplement: S3 Fig — The stool samples were normalized. The X and Y axis represent the stress level on a scale from 1–10. In gray is the confidence interval. The Pearson correlation coefficient is labeled “r” and the p value is labeled as “p”. (TIF) [file pone.0322530.s003.tif]

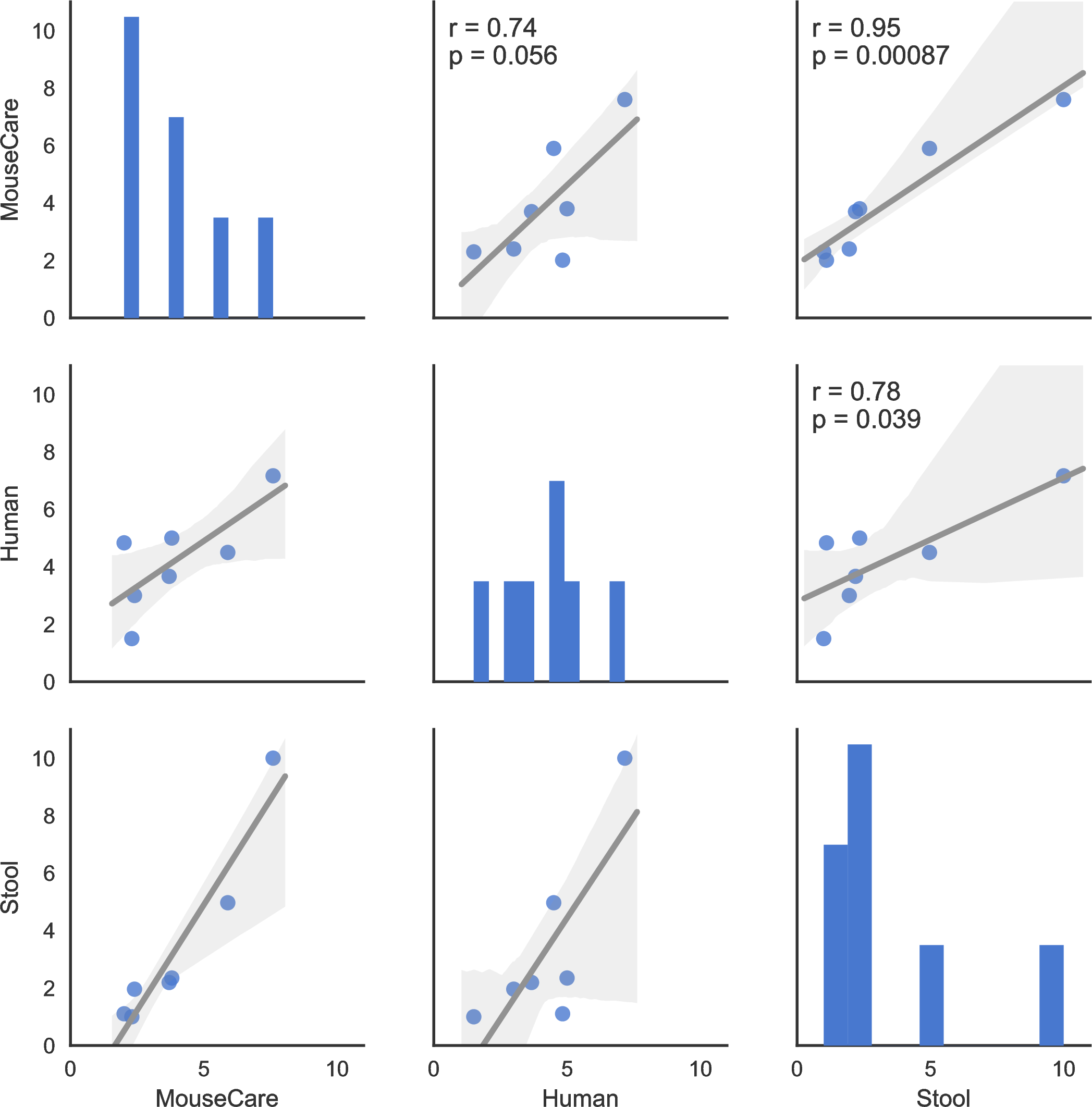

Supplement: S4 Fig — A) Numeric results of the Monte Carlo simulation shown in Fig 4D. B) Visual representation of the Performance comparison three groups. (TIF) [file pone.0322530.s004.tif]
